# Exploration of abnormal dynamic spontaneous brain activity in patients with high myopia *via* dynamic regional homogeneity analysis

**DOI:** 10.3389/fnhum.2022.959523

**Published:** 2022-08-05

**Authors:** Yu Ji, Qi Cheng, Wen-wen Fu, Pei-pei Zhong, Shui-qin Huang, Xiao-lin Chen, Xiao-rong Wu

**Affiliations:** Department of Ophthalmology, The First Affiliated Hospital of Nanchang University, Nanchang, China

**Keywords:** high myopia, brain function, brain region, resting-state functional magnetic resonance imaging, dynamics regional homogeneity

## Abstract

**Aim:**

Patients with high myopia (HM) reportedly exhibit changes in functional brain activity, but the mechanism underlying such changes is unclear. This study was conducted to observe differences in dynamic spontaneous brain activity between patients with HM and healthy controls (HCs) *via* dynamic regional homogeneity (dReHo) analysis.

**Methods:**

Resting-state functional magnetic resonance imaging (rs-fMRI) scans were performed on 82 patients with HM and 59 HCs who were closely matched for age, sex, and weight. The dReHo approach was used to assess local dynamic activity in the human brain. The association between mean dReHo signal values and clinical symptoms in distinct brain areas in patients with HM was determined *via* correlation analysis.

**Results:**

In the left fusiform gyrus (L-FG), right inferior temporal gyrus (R-ITG), right Rolandic operculum (R-ROL), right postcentral gyrus (R-PoCG), and right precentral gyrus (R-PreCG), dReHo values were significantly greater in patients with HM than in HCs.

**Conclusion:**

Patients with HM have distinct functional changes in various brain regions that mainly include the L-FG, R-ITG, R-ROL, R-PoCG, and R-PreCG. These findings constitute important evidence for the roles of brain networks in the pathophysiological mechanisms of HM and may aid in the diagnosis of HM.

## Introduction

High myopia (HM), a common ophthalmic disease, is the state of myopia with a refractive error of -6 diopters or worse (Flitcroft et al., 2019). In East Asia, approximately 80–90% of young people have myopia, and one-fifth of these people have HM (Wu et al., 2016). It is estimated that, by 2050, there will be 938 million individuals with HM (9.8% of the global population) (Holden et al., 2016). High academic pressures and limited time outdoors are regarded as key risk factors for myopia (Morgan et al., 2021). The high prevalence of myopia leads to an increased incidence of HM because of the relationship between these two diseases (Morgan et al., 2018). Excessive axial elongation is the most important pathological change in patients with HM; it can cause retinal detachment, choroidal neovascularization, macular hemorrhage, and retinal ischemia, all of which lead to impaired visual function (Piao et al., 2021). In clinical practice, resting-state functional magnetic resonance imaging (rs-fMRI) has recently received considerable attention. Some studies have shown that patients with HM exhibit changes in brain function, mainly in terms of cognitive function (Zhang et al., 2020), but the differences in dynamic spontaneous brain activity between patients with HM and healthy controls (HCs) remain unknown.

Resting-state functional magnetic resonance imaging is an emerging neuroimaging modality that provides a new non-invasive technique to study the relationship between spontaneous brain activity and clinical manifestations. Compared with other fMRI methods, rs-fMRI has the advantages of direct signal acquisition and the detection of functional regions in various patient populations (Smitha et al., 2017). Patients with HM reportedly exhibit significantly decreased functional connectivity (FC) between the supramarginal gyrus and rostrolateral prefrontal cortex, as well as between the ventral attention and frontoparietal control networks (Zhai et al., 2016). Huang et al. (2016) demonstrated that the amplitude of low-frequency fluctuation (ALFF) in the bilateral inferior frontal gyrus was considerably lower in patients with HM than in HCs. The above studies provide a neuroimaging basis for a better understanding of attentional control problems in patients with HM. Nevertheless, most rs-fMRI studies show that functional brain activity is stationary throughout the resting scan, but they overlook the time-dependent nature of spontaneous neuronal activity fluctuation in the brain (Calhoun et al., 2014; Liao et al., 2015, 2019). Regional homogeneity (ReHo) can only reflect the static characteristics of human spontaneous brain activity, which contradicts the notion that resting-state spontaneous neurocerebral activity has time-dependent dynamic characteristics. Thus, there has been an increasing focus on dynamic processes in spontaneous brain activity. Recently, dynamic regional homogeneity (dReHo) has been used in studies of various diseases to investigate the dynamic variability of spontaneous neuronal brain activity.

Dynamic regional homogeneity is a commonly used analysis in rs-fMRI, which can show the dynamic temporal consistency of spontaneous brain activity between neighboring voxels, describe similarities in local brain activity, and explore the functional coordination of spontaneous neural activity (Zang et al., 2004). Dynamics amplitude of low frequency fluctuation (dALFF) is an analytical method that combines the ALFF and a sliding window (Cui et al., 2020); this method measures the intensity of low-frequency oscillation in spontaneous neural activity, which can represent the intensity of neural activity in a single acceleration and demonstrate excitability in specific regions of the cerebral cortex (Zang et al., 2007). Thus far, ALFF technology has been used to study functional changes in spontaneous brain activity in patients with HM (Huang et al., 2016). In contrast to ReHo, dReHo involves the use of a sliding window method; areas with large fluctuations in dReHo are generally functional centers of the brain (Hutchison et al., 2013). dReHo has been used in studies of neuropsychiatric diseases, such as brain networks involved in bipolar disorder, clinical depression, trigeminal neuralgia, and other diseases (Yan et al., 2019; Sun et al., 2021). To our knowledge, no study has explored dReHo abnormalities in patients with HM. There is increasing evidence that patients with HM have a greater cognitive impairment as compared to HCs (Zhang et al., 2020). Here, we investigated whether dReHo values differ between patients with HM and HCs, which may be related to the cognitive changes caused by HM.

## Participants and methods

### Participants

From August 2021 to December 2021, 82 patients with HM and 59 HCs were examined in the Department of Ophthalmology at Nanchang University’s First Affiliated Hospital. For each participant, age, sex, and educational background were all met. People with brain disorders were excluded based on their clinical findings and physical assessment. All participants were examined in the same clinic and provided written informed consent to participate in the study. All procedures were conducted in accordance with the Declaration of Helsinki, and ethical approval was granted by the Nanchang University’s First Affiliated Hospital’s Medical Ethics Committee (Jiangxi Province, China).

The inclusion criteria for patients with HM were the binocular vision of –6 diopters or worse; corrected decimal visual acuity better than 1.0; and the completion of MRI-related tests, optical coherence tomography, ultrasonography, and other ophthalmic examinations. The exclusion criteria for patients with HM were binocular vision better than -6 diopters, retinal detachment, maculopathy, choroidal neovascularization, retinal pigment epithelial disease, history of ocular trauma or ophthalmic surgery, neurological disease, and/or cerebral infarction.

Healthy controls were randomly selected from Nanchang City according to their age, sex, and educational background. The inclusion criteria for HCs were no ocular disease; no major illness (e.g., neurological illness or cerebral infarction); uncorrected decimal visual acuity better than 1.0; and the completion of MRI-associated tests, optical coherence tomography, ultrasonography, and other ophthalmic examinations.

### fMRI data acquisition

A 3-T Trio Magnetic Resonance Imaging Scanning System (Trio Tim, Siemens Medical Systems, Erlangen, Germany) was used to collect all MRI data. During image acquisition, we asked participants to close their eyes, minimize motion, and avoid falling asleep. We also asked participants to use earplugs to reduce the effects of head motion and machine noise during scanning. The following three-dimensional high-resolution T1WI parameters were used: repetition time = 1,900 ms, echo time = 2.26 ms, thickness = 1, no intersection gap, acquisition matrix = 256 × 256, field of view = 240 × 240 mm^2^, and flip angle = 12°.

### fMRI data processing

In the brain imaging data processing and analysis toolbox (Data Processing and Analysis of Brain Imaging; DPABI),^1^ the sliding time window method was used to calculate the dReHo index. In accordance with a previously described method, the minimum window length of the sliding time window was set at ≥ 1/*f*_min_ (the minimum frequency of the time series) because a shorter window length would increase the risk of false fluctuations in the time series (Leonardi and Van De Ville, 2015).

### Sliding time window analysis

In this study, the window length was 30 repetition time (TR) and the step size was 1 TR. In each time window, the ReHo indices of all brain voxels were calculated. Next, the standard deviations (SDs) of these ReHo brain maps were calculated to characterize the dynamics of ReHo. Finally, the smoothing kernel was set at 8 × 8 × 8 mm^3^ to smooth dReHo images.

### Statistical analysis

Two-sample *t*-tests were performed on the fMRI data using SPM8 software (two-tailed voxel-level: *p* < 0.01, glomerular filtration rate (GRF) correction, cluster-level: *p* < 0.05), which allows the assessment of differences in zReHo maps between two groups *via* the GRF method. The GRF method was used to compensate for multiple comparisons and to adjust for age and sex.

Based on the dReHo calculations, some brain regions showed differences in signals between patients with HM and HCs. Thus, the mean dReHo value in each region was obtained by averaging all voxels within that region.

## Results

### Demographics

This study contained 82 patients with HM (43 men and 39 women, mean age 26.53 ± 5.291 years) and 59 HCs (24 men and 35 women, mean age 25.67 ± 3.102 years). Demographic characteristics are shown in Table 1.

**TABLE 1:** Demographic characteristics of patients with HM and HCs.

Characteristic	HM	HC
Sex (male/female)	43/39	24/35
Age (years)	26.53 ± 5.291	25.67 ± 3.102

HM, high myopia; HC, healthy control.

### Group differences in dynamic regional homogeneity

**FIGURE 1:**
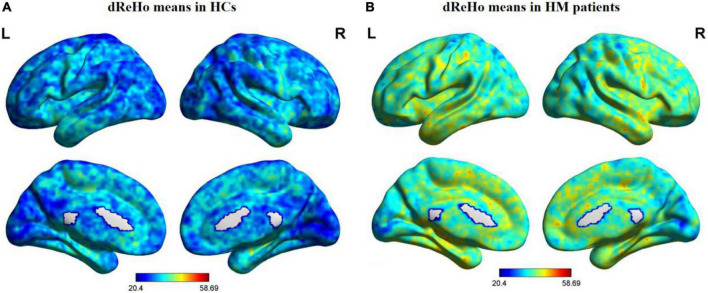
Distribution patterns of dReHo values are observed at the group level in HCs and patients with HM. Note: One-sample *t*-tests were used to compare dReHo maps between HCs **(A)** and patients with HM **(B)** (*p* < 0.01). HCs, healthy controls; HM, high myopia; dReHo, dynamic regional homogeneity analysis; L, left; R, right.

**FIGURE 2:**
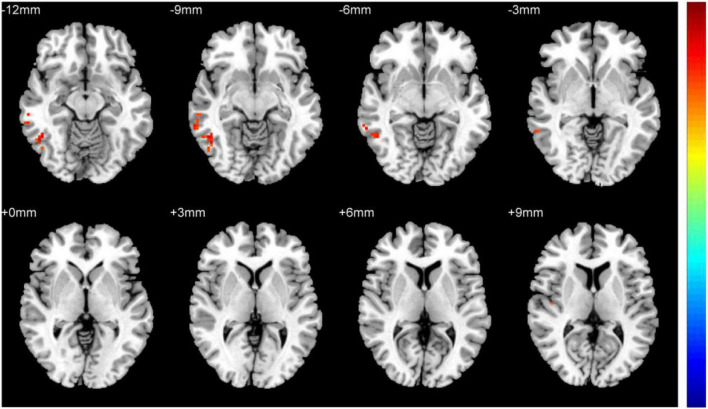
Comparison of differences in dReHo values between HCs and patients with HM. Significant differences in dReHo values are observed in the L-FG, R-ITG, R-ROL, R-PoCG, and R-PreCG. L-FG, left fusiform gyrus; R-ITG, right inferior temporal gyrus; R-ROL, right Rolandic operculum; R-PoCG, right postcentral gyrus; R-PreCG, right precentral gyrus.

Figure 1 shows comparisons between the HM and HC groups. dReHo values in the left fusiform gyrus (L-FG), right inferior temporal gyrus (R-ITG), right Rolandic operculum (R-ROL), right postcentral gyrus (R-PoCG), and right precentral gyrus (R-PreCG) were significantly higher in patients with HM than in HCs. The mean values of differences in dReHo between the two groups are shown in Figure 2. The mean differences in dReHo values between HM patients and HCs are shown in Table 2.

**TABLE 2:** Mean differences in dReHo values between patients with HM and HCs.

Brain region	BA	Peak t-score	MNI coordinates (x, y, z)	Cluster size (voxels)
L-FG	20	3.4825	–33, –39, –24	13
R-ITG	–	3.4744	66, –39, –15	24
R-ROL	–	4.5164	42, –24, 15	18
R-PoCG	–	4.6369	54, –12, 39	40
R-PreCG	–	4.6523	18, –27, 78	38

Two-tailed voxel-level: p < 0.01, GRF correction, cluster-level: p < 0.05. BA, Brodmann area; MNI, Montreal Neurological Institute; L-FG, left fusiform gyrus; R-ITG, right inferior temporal gyrus; R-ROL, right Rolandic operculum; R-PoCG, right postcentral gyrus; R-PreCG, right precentral gyrus.

## Discussion

To our knowledge, this is the first study to use the dReHo method for the estimation of the effect of HM on dynamic spontaneous brain activity. The dReHo method can help to provide a greater understanding of HM-related functional remodeling in the brain. We found that patients with HM had significantly increased dReHo values in the L-FG, R-ITG, R-ROL, R-PoCG, and R-PreCG, which suggested a degree of synchronization in dynamic spontaneous brain activity among those regions. These changes could be linked to the activity of various areas in the brain.

The L-FG has a significant impact on language morphological processing (Zou et al., 2015), lexical processing of real words, and grapheme-to-phoneme processing of pseudo-words (Krishnamurthy et al., 2019). The FG has spatially separated regions: the right side is more sensitive to facial recognition, while the left side is more sensitive to language recognition (Harris et al., 2016). Zu et al. (2022) found that patients with persistent generalized tonic-clonic seizures (GTCS) had increased ALFF values in the bilateral FG, which may be useful as a novel neurological marker for persistent seizures in patients with GTCS. Jung et al. (2021) reported that L-FG volume was associated with the recognition of emotional intensity and facial emotion by people with schizophrenia spectrum psychosis. Chen B. et al. (2021) revealed that patients with late-life depression (LLD) and odor identification (OI) dysfunction showed significantly increased ReHo values in the L-FG. Therefore, increased ReHo values in the L-FG may indicate an increased risk of OI dysfunction in patients with LLD. Moreover, a previous study showed that patients with HM have decreased FC in the L-FG, which may cause differences in tactile function between patients with HM and HCs (Wu et al., 2020). Consistent with the previous findings, we demonstrated that patients with HM had significantly increased dReHo values in the L-FG, which suggests that behaviors in this brain area are reinforced. Thus, we speculate that increased dReHo values in the L-FG are related to dysfunctional language morphological processing in patients with HM; in such patients, the increased dReHo values in the L-FG may compensate for the decline in language morphological processing function.

The R-ITG plays an important role in higher cognitive functions, such as visual and language comprehension, as well as emotional regulation (Lin et al., 2020). Wei et al. (2021) demonstrated that FC in the ITG was increased in patients who had migraine without aura. They noted that the ITG is regarded as a component of the default mode network (DMN) (Liu et al., 2017) and has been associated with worsening pain. Li H. et al. (2021) reported that ReHo values in the bilateral ITG were greater in patients with obstructive sleep apnea (OSA). They showed that increased ReHo values in the bilateral ITG were positively correlated with the apnea-hypopnea index (AHI). Yuan et al. (2016) found that patients with amnestic moderate cognitive impairment (aMCI) had considerably lower ReHo values in the ITG when compared with HCs. Such a change in ReHo values could serve as a sensitive functional imaging biomarker for aMCI. Furthermore, Tu et al. (2018) revealed that ReHo values in the R-ITG were significantly higher in alcohol-dependent individuals than in HCs; notably, this region is responsible for the representation and detection of complex object features. In support of the previous findings, our study showed that patients with HM had significantly greater dReHo levels in the R-ITG. Thus, we presume that increased dReHo values in the R-ITG may be indicative of deficits in higher cognitive functions and vision in patients with HM; they might represent a functional activity to compensate for such deficits.

The R-ROL is involved in the processing of integrated exteroceptive-interoceptive information (Blefari et al., 2017). During the perception of pleasant auditory information, motor-related circuitry in the ROL may facilitate the formation of vocal representations (Koelsch et al., 2006). Zhang et al. (2021) demonstrated that individuals with addiction-related disorders had common decreases in gray matter (GM) volume in the R-ROL. They discovered similar structural changes in the prefrontal and insular areas of the brain among patients with different subtypes of addiction. Furthermore, Wollman et al. (2017) found that length of opioid use was negatively associated with GM in the R-ROL, which suggested that opioid addiction could lead to the disintegration of strongly overlapping structural and functional systems. Additionally, Li L. et al. (2021) reported that in the R-ROL, patients with Crohn’s disease (CD) had considerably increased FC intensity. This could be a positive feedback mechanism for increased sensitivity to visceral sensory information, which modulates the brain’s response to such information and may exacerbate inflammation. In a separate study, Li J. et al. (2021) revealed that, compared with HCs, patients with Parkinson’s disease (PD) had considerably lower ReHo values in the R-ROL; this suggested a negative relationship between ReHo values and cognition. Indeed, the R-ROL has been associated with the severity of alterations in psychological domains, such as apathy, despair, and anxiety (Sutoko et al., 2020). Expanding upon the prior findings, this study showed that patients with HM had considerably higher dReHo values in the R-ROL, which suggests that behaviors in this brain area are reinforced. Thus, we speculate that HM leads to increased R-ROL activity, which can cause deficits in the processing of integrated exteroceptive-interoceptive signals in patients with HM; the increased dReHo values in the R-ROL may compensate for the reduced integration of exteroceptive-interoceptive signals.

The R-PoCG contributes to the processing of sensory data from various parts of the body (Kropf et al., 2019). Additionally, the PoCG is the main sensory reception area for touch, proprioception, pain, and temperature (Cauda et al., 2009). Hu et al. (2018) demonstrated that patients with HM had significantly decreased degree centrality (DC) values in the R-PoCG, which confirmed sensorimotor network (SMN) remodeling in such patients. Huang et al. (2016) reported that patients with HM showed higher ALFF values in the R-PoCG. Furthermore, Chen W. et al. (2021) found that patients with thyroid-associated ophthalmopathy (TAO) had significantly decreased DC values in the R-PoCG; the duration of illness was negatively correlated with DC values in the PoCG. Additionally, Zhen et al. (2018) revealed that patients with aMCI had considerably higher ReHo values in the R-PoCG. Such localized changes in FC imply that these networks simultaneously experience functional deficiencies and compensation. In the present study, we found that patients with HM had considerably higher dReHo values in the R-PoCG, which suggests that behaviors in this brain area are reinforced. Thus, we speculate that HM leads to increased R-PoCG activity, which may broadly impair the processing of sensory information in patients with HM. However, the increased dReHo value in the R-PoCG may compensate for this reduced sensory processing ability.

The R-PreCG plays a critical role in sensorimotor processing (Desmurget et al., 2014). Tong et al. (2021) demonstrated that patients with iridocyclitis had significantly decreased ALFF in the R-PreCG. Wang et al. (2021) reported that patients with primary angle-closure glaucoma showed significantly decreased fractional ALFF in the R-PreCG. Furthermore, Jiang et al. (2021) revealed that, compared with HCs, patients with diabetic optic neuropathy had significantly higher ALFF values in the R-PreCG; they suggested that ALFF could be used to distinguish patients with diabetic optic neuropathy from individuals without the disease. Additionally, Duan et al. (2014) found that patients with neuromyelitis optica (NMO) had significantly decreased white matter volumes in the R-PreCG, which indicated the presence of subtle white matter damage to the motor, visual, and cognitive systems in such patients. Our present findings indicated that the HM group had considerably higher dReHo values in the R-PreCG, which suggests that behaviors in this brain area are reinforced. Therefore, we speculate that increased dReHo values in the R-PreCG lead to deficits in the sensorimotor processing of patients with HM; the increased dReHo values in the R-PreCG may compensate for this reduced sensorimotor processing ability.

Importantly, this study had some limitations. First, patients with HM in this trial were mostly young adults. Second, the data were frequently affected by some unavoidable factors in the fMRI environment (e.g., heartbeat, muscle beat, and respiratory motion). Finally, the patients had various lengths of HM history, which may have affected the accuracy of the findings. In future studies, we plan to focus on including participants of all ages and improving the test environment.

## Conclusion

Our results suggest that, compared with HCs, patients with HM have altered dReHo values in various brain regions, which implies that HM causes extensive changes in dynamic spontaneous brain activity; these changes presumably lead to the corresponding clinical manifestations. Our findings offer new insights into the causes and neural mechanisms of HM, and they may serve as guidance for its diagnosis.

## Data availability statement

The raw data supporting the conclusions of this article will be made available by the authors, without undue reservation.

## Ethics statement

The studies involving human participants were reviewed and approved by Nanchang University’s First Affiliated Hospital’s Medical Ethics Committee. The patients/participants provided their written informed consent to participate in this study. Written informed consent was obtained from the individual(s) for the publication of any potentially identifiable images or data included in this article.

## Author contributions

YJ was responsible for writing the manuscript. QC was in charge of proofreading and refining the manuscript’s wording. W-WF, P-PZ, S-QH, and X-LC contributed to data collection and statistical analyses. YJ and QC designed the protocol and contributed to the MRI analysis. YJ, QC, and X-RW designed the study, oversaw all clinical aspects of study conduct, and manuscript preparation. All authors contributed to the article and approved the submitted version.

## Footnotes

1
http://rfmri.org/dpabi
